# Evaluation of the *in vitro* acaricidal activity of Chinese herbal compounds on the poultry red mite (*Dermanyssus gallinae*)

**DOI:** 10.3389/fvets.2022.996422

**Published:** 2022-09-27

**Authors:** Yichen Jian, Huizhen Yuan, Dongliang Li, Qing Guo, Xiaoying Li, Sumei Zhang, Changshen Ning, Longxian Zhang, Fuchun Jian

**Affiliations:** ^1^College of Animal Veterinary Medicine, Henan Agricultural University, Zhengzhou, China; ^2^International Joint Research Center for Animal Immunology of China, Zhengzhou, China; ^3^Hennan Hemu Animal Pharmaceutical Co., Ltd., Zhengzhou, China

**Keywords:** Chinese herbal compound, ethanol extract, poultry red mite, content determination, toxic effects

## Abstract

The poultry red mite *Dermanyssus gallinae* is an economically important pest in poultry farms worldwide, but an effective treatment option is lacking. The current study determined the effectiveness of six Chinese herbal medicines [*Syzygium aromaticum* (clove), *Hibiscus syriacus* (Hibiscus), *Illicium verum* (star anise), *Leonurus artemisia* (motherwort), *Cinnamomum cassia* (cinnamon), and *Taraxacum* sp. (dandelion)] against *D. gallinae*. Alcohol extracts were prepared *via* the solvent extraction method and the phenol, flavonoid, and tannin contents were determined. These active components were highest in *S. aromaticum* and lowest in *H. syriacus, I. verum*. No tannin content was detected in *L. artemisia*. All extracts showed contact toxicity against *D. gallinae* at a test concentration of 1 g/mL, with *S. aromaticum* and *L. artemisia* resulting in 100% mortality. *S. aromaticum, L. artemisia*, and *I. verum* showed the best efficacy (LC_50_ 0.159, 0.200, and 0.292 g/mL, respectively). Different combinations of extracts showed an additive effect of *I. verum* LC_90_ + *L. artemisia* LC_90_. The acaricidal efficacy of this combination was tested against different developmental stages of *D. gallinae*, being most efficacious against nymphal and larval *D. gallinae*, with a corrected mortality rate of 100%. However, inhibition of egg hatching was only 53.69%. Taken together, these results highlight *I. verum* LC_90_ + *L. artemisia* LC_90_ as a promising compound with severe contact toxicity against *D. gallinae*. Given the wide cultivation of these species and their extensive use in foodstuffs and cosmetics as flavors and fragrances, they could be a cheap, readily available ecofriendly alternative to pesticides currently used in poultry farms.

## Introduction

The poultry red mite (PRM), *Dermanyssus gallinae* (De Geer, 1778) (Acari, Dermanyssidae), is a common ectoparasitoid that feeds on poultry and human blood, and is highly prevalent in poultry farms worldwide, causing annual losses of millions of Euros (1). PRM are tiny, hiding in cracks in the walls of poultry houses and chicken coops during the day, and sucking blood from the host at night. PRM infection can lead to restlessness, pecking of feathers, skin inflammation, anemia, and slow growth, and can also affect egg production and egg quality (2, 3). PRM infection outbreaks can not only cause serious harm to the poultry industry, but also pose a threat to human health. In recent years, there has been a series of reports of infections in humans caused by PRM bites (4, 5), such as rash, dermatitis, urticaria, and other skin diseases. In addition, PRM have been reported to bite cats and dogs (6, 7). Moreover, PRM are also a transmitter of more than 20 species of pathogen (8–10), including *Escherichia coli, Streptomyces* spp., *Staphylococcus* spp., *Yersinia, Listeria*, and *Pasteurella* spp., as well as eastern equine encephalitis virus, Venezuelan equine encephalitis virus, and alphavirus (11, 12). At present, the prevention and control of PRM mainly rely on chemical drugs. However, the long-term use of chemical drugs, such as organophosphorus and ivermectin, not only leads to PRM resistance, but also has a significant impact on the environment and egg production (13–16). Therefore, there is a need to develop new compounds that are not harmful to humans or the environment, that do not negatively impact the quality of eggs and have good acaricidal effects. The use of Chinese herbal medicines is becoming more popular in the poultry industry because of their natural safety, little if any development of drug resistance, and residue characteristics. Chinese herbal medicines have antibacterial and antiviral effects, as well as enhancing immunity and other functions, and have been developed and applied in the prevention and treatment of modern animal parasitic diseases (17). Although botanical pesticide research has mainly focused on mosquito and ticks (18), studies on other arthropods of medical and veterinary importance are still in the preliminary phase. Several botanical pesticides are currently used in arthropod pest management, such as products based on the neem tree (*Azadirachta indica*). Neem extracts are reported to have toxic effects against 200 species of arthropod pests (19). Herbs can produce a broad range of secondary metabolites, such as terpenoids, polyacetylenes, sugars, flavonoids, and alkaloids, which can act as antifeedants and repellents. In addition, they can also suppress acetylcholinesterase activity, negatively impacting the nervous system (20–23). They can also act on other targets in the nervous system, such as nicotinic acetylcholine receptors (nAChR), octopamine receptors, tyramine receptors, sodium channels, and γ-aminobutyric acid (GABA)-gated chloride channels (24, 25).

Thus, the current study evaluated the toxic effects of Chinese herbal medicines on *D. gallinae*, namely clove, Hibiscus, star anise, motherwort, cinnamon, and dandelion, which were selected based on previous studies (26–28). The toxicity of these Chinese herbal medicines was evaluated through contact assays on adult mites with compound synergy assays against different stages. The herbs with best acaricidal activity were screened out through contact assays, and the compound synergistic effect of the selected herbal medicine was tested. Finally, the best herbal was screened out according to the acaricidal effect of different developmental stages.

## Materials and methods

### Herbal preparations

The experimental plants [clove (flower bud), cinnamon (bark), hibiscus (flowers), dandelion (whole plant), and star anise (ripe fruit), motherwort (whole plant)] were all purchased from Tongrentang Chinese Herbal Medicine Wholesale Store (Beijing, China). A positive control (100 mg/mL ivermectin) was purchased from Henan Anjin Biotechnology (Zhengzhou, Henan, China). Anhydrous ethanol (analytical grade) was purchased from Fuyu Fine Chemical (Tianjin, China). A negative control (0.9% sodium chloride) was purchased from Henan Kelun Pharmaceutical (Anyang, Henan, China). Blank control distilled water was produced by the Parasitology Laboratory, Henan Agricultural University.

### Ethanol extraction of herbal medicines

For each herb (see above), 50 g of the dried tissues was crushed and passed through 30-mesh screens. Next, 200 mL of a 90% ethanol solution was added; the samples were soaked for 1 week and then filtered through six layers of gauze. The residue was then added to 100 mL of a 90% ethanol solution and soaked again for 24 h. This solution was filtered through six layers of gauze, and the two filtrates were then combined. The filtrate was centrifuged at 3,000 rpm for 10 min, and the ethanol was evaporated from the supernatant in a boiling water bath to concentrate it into a paste. This paste was diluted to 50 mL with 0.9% sodium chloride; thus, 50 g of each herb was used to make 50 mL of experimental insecticide. Each insecticide was stored at 4°C for later use.

### Determination of total phenolic compounds, flavonoids, and tannins in the herbal alcohol extracts

The total phenolic compound content of the six herbal alcohol extracts was measured using the Folin-Ciocalteu method (29). First, a standard solution of gallic acid (0, 0.05, 0.1, 0.15, 0.2, 0.25, and 0.3 mg/mL) was prepared. Then, 1 mL of each extract, 3 mL of distilled water, 1 mL of Folin-Ciocalteu phenol reagent and 4 mL of 7.5% sodium carbonate solution were combined and diluted to 10 mL with distilled water in a volumetric flask (10 mL). The mixture was shaken well and allowed to stand for 1.5 min at 30°C in darkness. Absorbance for the test and standard solutions was read at 760 nm using distilled water for zero adjustment. All samples were replicated six times. Gallic acid solutions (0–0.3 mg/mL) were used to generate a standard curve (*r*^2^ = 0.9998). The gallic acid content was presented in terms of mg/mL of gallic acid in the sample.

The Ruslin method was used to determine the total flavonoid content (30). First, a standard solution of rutin (0, 0.2, 0.4, 0.6, 0.8, 1.0, and 2.0 mg/mL) was prepared. To each 0.1 mL of herbal alcohol extract, 0.3 mL sodium nitrite solution with a concentration of 5% was added; the mix was shaken well and left to stand for 6 min. Then, 0.3 mL aluminum nitrate solution with a concentration of 10% was added and the mix was again shaken well and left to react for 15 min away from light. Next, 4 mL 4% sodium hydroxide solution was added, followed by a constant volume of 80% ethanol solution to the scale line. After 15 min of mixing and incubation at room temperature, the absorbance of the reaction mixtures was determined at 510 nm against a blank. Rutin solutions (0–2 mg/mL) were used to generate a standard curve (*r*^2^ = 0.9995). The total flavonoid assay was repeated six times for each extract.

Use Tannin Acid Content Assay kit, Micromethod (Sangon Biotech, Shanghai, China) instructions for determination. All samples were carried out in 6 copies. Tannin content solutions (0–10 mg/mL) were used to generate a standard curve (*r*^2^ = 0.9994). The tannin content was presented in terms of mg/mL of tannic acid in each sample.

### Acquisition of mites

On June 6, 2021 a suspected case of chicken mite infestation occurred at an egg farm in Tongxu County, Kaifeng City, Henan Province, China, with dense, fast-moving mites appearing in the chicken coop and surrounding cracks in the wall. A small brush was used to collect mites from the structure of the chicken coops, feed and water troughs, chickens body surface, and so on, and there were transferred into a sealed bag, which was taken to the Parasitology Laboratory of Henan Agricultural University. All experiments were approved by the Animal Welfare and Research Ethics Committee of the College of Animal Medicine, Henan Agricultural University (Permit No: HNND2021060601). Under a stereomicroscope, using the identification method of Di Palma (31), the morphology of the mouth organs, horns, and vent region of the mites was examined, and the molecular method of Chu was used for further identification (32). The final identification confirmed the presence of *D. gallinae*. According to the sequence comparative analysis of homology, the samples collected showed 100% homology with the *D. gallinae* LC034951.1 gene sequence from Japan. The collected mites were then stored in a 4°C refrigerator until use.


(1) $$\begin{array}{l} \text{Theoretical mortality rate of mixture }(\%)\text{ = }[\text{1 − }(\text{1 − mortality rate of each single dose test group})\;\\ \;\times \;(\text{1 − mortality rate of each single dose test group})] \end{array}$$

### Evaluation of the toxic effects of Chinese herbal medicine extracts

For each Chinese herbal medicine extract, the bottom of a 60 × 15 mm petri dish was lined with filter paper and 1 g/mL of extract was evenly added to the filter paper. The dish was then left for 24 h to allow for volatilization of the extract to form a drug film. Ten similar-sized *D. gallinae* of good physiological status were selected with a small brush, and placed each petri dish and left for 1 h. The mites were then transferred to a clean petri dish without a drug film. There were five replicates per extract per set of experiments. A treatment group containing no agent was set as a blank control, whereas the negative control dish contained 0.9% NaCl, and positive control contained 100 mg/mL ivermectin.

After exposure to the Chinese herbal medicine extract for 1 h, the mites were incubated in a constant temperature (25–30°C) and humidity (relative humidity 60–80%) incubator in a light: dark 12:12 h photoperiod (33). Their mortality was observed and recorded 48 h later. If a mite remained immobile after being continuously stimulated with a needle for 1 min, it was considered to be dead.

### Method for determining synergistic effects between Chinese herbal medicines

The adopted a method to determine whether the mixed use of two plant sources has synergistic effects using Equation (1):

The synergistic virulence index (Equation 2) was used to evaluate the combined effect of two agents:


(2) $$\begin{array}{l} (\text{c}\cdot \text{f})\text{ = }[(\text{actual death rate − theoretical death rate})\text{/ }\\ \text{ theoretical death rate}]\;\times \text{ 100} \end{array}$$

where c·f > 20 indicated a synergistic effect, c·f < −20 indicated antagonistic effects, and −20 <c·f < 20 indicated additive effects. Each pair of Chinese herbal medicine extracts was analyzed using both approaches.

### Statistics and analysis

The experimental data were sorted with Excel, and the percentage mortality of each test group was calculated using Equation (3):


(3) $$\begin{array}{l} \text{mortality (\%) = (no. dead mites/total no. tested mites) }\\ \text{ × 100\%} \end{array}$$

The mortality in each treatment group was corrected to take into account control mortality using Abbott's formula (Equation 4) (34):


(4) $$\begin{array}{l} \text{corrected mortality (\%) = [1 − \% dead mites in the treated plate/\% dead mites in the untreated control plate] }\\ \text{ × 100\%} \end{array}$$

If the mortality rate was <5%, there was no adjustment for mortality.

The egg-hatching rate was calculated using Equation (5):


(5) $$\begin{array}{l} \text{ Egg − hatching rate = }(\text{number of eggs hatched/ }\\ \text{ total number of eggs})\;\times \text{ 100}\% \end{array}$$

Data were expressed as the mean ± standard deviation. SPSS v.20.0 was used to analyze the date using one-way ANOVA. *P* < 0.05 indicated statistically significant differences between the treatment and control groups. Coefficient of determination (*r*^2^) and regression equations were calculated by linear regression. The median lethal concentration (LC_50_) and 95% confidence interval (95% CI) were calculated using the Probit algorithm. Prism v.8.0 software was used to draw the figures (GraphPad, San Diego, CA, USA).

## Results

### Total phenol, flavonoid, and tannin contents of the Chinese herbal medicine alcohol extracts

Table 1 summarizes the results from the quantitative determination of the tannin and flavonoid content of each plant extract and their respective total phenol content. Clove contained the highest levels overall (70.78 mg/mL), and of total phenols (66.50 mg/mL), flavonoids (3.97 mg/mL), and tannins (0.31 mg/mL), followed by star anise (64.88 mg/mL), total phenols (64.00 mg/mL), flavonoids (0.76 mg/mL), tannins (0.12 mg/mL); cinnamon (45.45 mg/mL), total phenols (45.00 mg/mL), flavonoids (0.41 mg/mL), and tannins (0.04 mg/mL); dandelion (21.58 mg/mL), total phenols (21.50 mg/mL), flavonoids (0.04 mg/mL) and tannins (0.04 mg/mL). Hibiscus contained the lowest levels overall (7.31 mg/mL), with low total phenols (7.00 mg/mL), flavonoids (0.24 mg/mL), and tannins (0.07 mg/mL), whereas motherwort was slightly higher overall (16.90 mg/mL), including total phenols (16.70 mg/mL) and flavonoids (0.20 mg/mL), and but contained no tannins detectable by the methods used.

**Table 1:** Active components of alcohol extracts from six Chinese herbal medicines.

**Active component**	**Chinese herbal species (mg/mL)**					
	**Motherwort**	**Dandelion**	**Clove**	**Star anise**	**Cinnamon**	**Hibiscus**
Total phenolics	16.70	21.50	66.50	64.00	45.00	7.00
Total flavonoids	0.20	0.04	3.97	0.76	0.41	0.24
Condensed tannins	0	0.04	0.31	0.12	0.04	0.07
Total content	16.90	21.58	70.78	64.88	45.45	7.31

### Effects of herbal extracts on mite mortality

**Figure 1:**
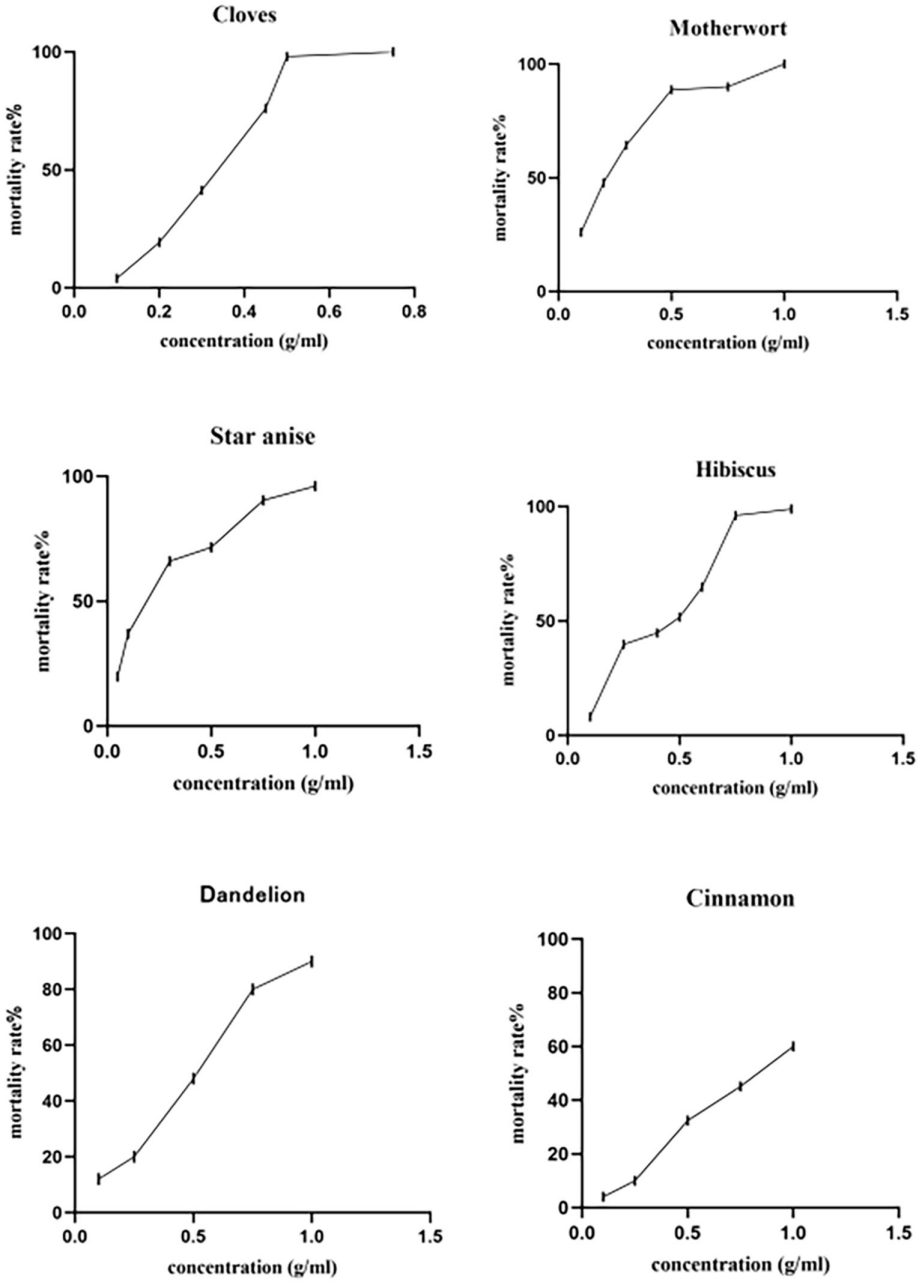
Contact toxicity of alcohol extracts of Chinese herbal medicines against adult *Dermanyssus gallinae*.

At a concentration of 1 mg/mL, the tested herbs showed different degrees of toxicity against adult *D. gallinae* (Table 2), with clove and motherwort resulting in 100% adjusted mortality, followed by hibiscus (98.82%), star anise (96.00%), and dandelion (90.00%). By contrast, cinnamon showed weak toxicity with an adjusted mortality of 60.00%. In terms of LC_50_ and LC_90_, star anise LC_50_ (0.159 g/mL) and motherwort LC_50_ (0.200 g/mL) had the strongest effects. By contrast, the LC_50_ of clove, hibiscus, and dandelion were 0.292 g/mL, 0.388 g/mL, and 0.410 g/mL, respectively. The strongest LC_90_ effect was seen with clove (0.521 g/mL) and motherwort (0.622 g/mL), compared with star anise (0.886 g/mL), hibiscus (1.119 g/mL), and dandelion (1.290 g/mL). The LC_50_ and LC_90_ of cinnamon were 0.812 mg/mL and 3.163 mg/mL, respectively (Table 3 and Figure 1). Thus, given these levels of biological activity and resource costs, star anise, clove, and motherwort were selected for further study.

**Table 2:** Effect of alcohol extracts of Chinese herbal medicines on adult *Dermanyssus gallinae*.

**Treatment^A^**	**No. of adults tested**	**Adjusted mortality (%) after 48 h (mean ±S.D.)**
Clove	51	100.00 ± 0.00^a^
Motherwort	52	100.00 ± 0.00^a^
Hibiscus	51	98.82 ± 2.63^a^
Dandelion	50	90.00 ± 10.00^a^
Star anise	50	96.00 ± 8.94^a^
Cinnamon	50	60.00 ± 18.70^b^
100 mg/mL ivermectin (positive control)	50	100.00 ± 0.00^ac^
Negative control	50	0.00 ± 0.00^d^
Blank control group	50	0.00 ± 0.00^d^

^A^Test concentration of alcohol extracts of Chinese herbal medicines was 1.0 mL, the corrected mortality rate (%) was the average of five repetitions. Values followed by different letters indicate statistical significance (P < 0.01).

**Table 3:** Toxic effects of herbal alcohol extracts against adult *Dermanyssus gallinae*^A^.

**Treatment**	**LC_50_ (g/mL)**	**CI95%**	**LC_90_ (g/mL)**	**CI 95%**	***r* ^2^**	**Regression equation (*y*=)**
Star anise	0.159	0.122–0.199	0.886	0.656–1.352	0.863	29.523 + 75.349χ
Motherwort	0.200	0.166–0.230	0.622	0.523–0.790	0.834	33.52 + 75.68χ
Clove	0.292	0.211–0.376	0.521	0.400–0.987	0.860	−6.772 + 164.9χ
Hibiscus	0.388	0.131–0.680	1.119	0.651–43.318	0.916	5.151 + 102.095χ
Dandelion	0.410	0.208–0.738	1.290	0.722–12.308	0.967	1.024 + 94.184χ
Cinnamon	0.812	0.664–1.076	3.163	2.035–6.972	0.987	−3.005 + 64.091χ

^A^CI 95%, 95% confidence interval; LC_50_, Median lethal concentration (g/mL); LC_90_, 90% lethal concentration(g/mL); r^2^, correlation coefficient.

### Synergistic effects of Chinese herbal medicine alcohol extracts

The synergistic virulence index against *D. gallinae* adults of the six combinations of herbal extracts showed that star anise LC_50_ + motherwort LC_50_, star anise LC_50_ + motherwort LC_90_, and clove LC_50_ + motherwort LC_90_ had antagonistic effects, whereas star anise LC_90_ + motherwort LC_50_, clove LC_90_ + star anise LC_90_, and star anise LC_90_ + motherwort LC_90_ had additive effects. The mortality rate of *D. gallinae* adults treated with star anise LC_90_ + motherwort LC_90_ was 96.55%, whereas, with clove LC_90_ + star anise LC_90_, it was 87.64% (Table 4). Thus, based on the actual mortality rate, star anise LC_90_ + motherwort LC_90_ was selected for further study.

**Table 4:** Synergetic toxicity index (c·f value) of herbal alcohol extracts from Chinese herbal medicines against adult *Dermanyssus gallinae*^A^.

**Combination**	**No. of mites tested**	**Theoretical mortality (%)**	**Actual mortality (%; mean±S.D.)**	**c·f value**
Star anise Lc_50_ + motherwort Lc_50_	600	75	41.83 ± 10.04^a^	−44.22
Star anise Lc_50_ + motherwort Lc_90_	615	95	60.01 ± 6.76^b^	−36.83
Star anise Lc_90_ + motherwort Lc_90_	605	99	96.55 ± 2.80^c^	−2.47
Star anise Lc_90_ + motherwort Lc_50_	600	95	86.33 ± 3.80^d^	−9.12
Clove LC_90_ + star anise LC_90_	605	99	87.64 ± 5.00^d^	−11.47
Clove LC_90_ + motherwort LC_90_	600	99	64.49 ± 4.43^be^	−34.85
100 mg/mL ivermectin (positive control)	600	/	100.00 ± 0.00^cf^	/
Negative control	600	/	8.00 ± 13.03^g^	/

^A^Test concentrations of the above single doses were: Illicium verum Hook. f. LC_50_: 0.159 g/mL, I. verum Hook. f. LC_90_: 0.886 g/mL, Leonurus japonicus Houtt LC_50_: 0.20 g/mL, L. japonicus Houtt LC_90_: 0.622 g/mL, Syzygium aromaticum (L.) LC_90_: 0.521 g/mL; values followed by different letters within the same column are statistically significant (P < 0.01).

### Contact toxicity of combined Chinese herbal alcohol extracts against the development stages of *Dermanyssus gallinae*

**Figure 2:**
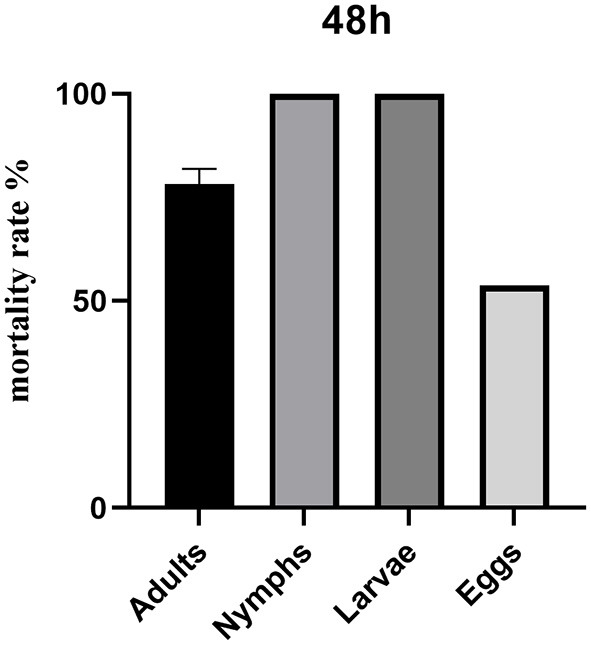
Contact toxicity after 48 h of treatment with star anise LC_90_ + motherwort LC_90_ against different development stages of *Dermanyssus gallinae*.

Star anise LC_90_ + motherwort LC_90_ was most effective against *Dermanyssus gallinae* nymphs and larvae, with a mortality rate of 100.00% (Table 5 and Figure 2). By contrast, the corrected mortality of adult *Dermanyssus gallinae* in response to star anise LC_90_ + motherwort LC_90_ was 78.16%, and the rate of egg-hatching inhibition was 53.69%.

**Table 5:** Contact toxicity of compound herbal medicines against developmental stages of *Dermanyssus gallinae*.

**PRM stage**	**Treatment**	**No. of samples**	**Mortality (%; mean ±S.D.)**
Adult	Star anise LC_90_+ motherwort LC_90_	600	78.16 ± 3.69^a^
	100 mg/mL ivermectin	600	100.00 ± 0.00^b^
	Negative control	600	8.00 ± 13.03^c^
Nymph	Star anise LC_90_+ motherwort LC_90_	602	100.00 ± 0.00^a^
	100 mg/mL ivermectin	600	100.00 ± 0.00^a^
	Negative control	600	8.00 ± 13.03^c^
Larva	Star anise LC_90_+ motherwort LC_90_	600	100.00 ± 0.00^a^
	100 mg/mL ivermectin	600	99.83 ± 0.37^a^
	Negative control	600	12.99 ± 5.08^b^
Egg	Star anise LC_90_+ motherwort LC_90_	103	53.69 ± 21.88^a^
	100 mg/mL ivermectin	100	38.48 ± 7.83^b^
	Negative control	100	73.33 ± 11.54^c^

^a,b,c^Values followed by different lowercase letters within the same column are statistically significant (P < 0.01).

## Discussion

Analysis of the composition of the six Chinese herbal medicine extracts showed total phenolics to be the main components, with flavonoids and tannins being present at low levels, if at all, similar to previous research (35). Thus, although the different herbals contained the same compounds, the relative amounts varied among species. This might be because climatic and edaphic conditions can have a strong influence on the composition of herbals (36–38). The total polyphenol, flavonoid, and condensed tannin contents of clove and star anise extracts were higher than in the other herbal extracts. Nevertheless, all plant extracts showed good acaricidal activity at 1 mg/mL, revealing for the first time the acaricidal effects of extracts of hibiscus and motherwort on adults of *D. gallinae*, with a mortality rate >98% (Table 2). In addition, star anise (LC_50_ = 0.159 g/mL) motherwort (LC_50_= 0.200 g/mL), and clove (LC_50_ = 0.292 g/mL) extracts showed significantly higher acaricidal activity compared with the other herbal extracts. Based on all parameters tested, the star anise and clove extracts exhibited the highest acaricidal activity, which could be due to the presence of a higher concentration of polar-soluble active molecule(s). Previous reports revealed that clove contains a volatile oil (15–20%) that mainly comprises eugenol (78–95%), acetyl eugenol (7.3%), and ß-caryophyllene (9%), star anise essential oil is characterized by phenylpropanoids (98.8 and 84.0%, respectively), with €-anethole (94.8 and 64.6%, respectively) as the predominant compound (27, 28). The authors investigated the efficacy of essential oil and fractions isolated from star anise and clove, respectively on *D. gallinae*, with *in vitro* test revealing a 100% mortality of clove at a concentration of 1.3 μg/m^2^; star anise was also found to have contact toxicity, with an LC_50_ of 59 μg/mL. There are also reports on the acaricidal and repellent properties of essential oils and extracts from other plant species against *D. gallinae*. for example, George et al. showed that thyme and cade oil are effective acaricides against *D. gallinae* when tested over a 24-h period (39). Nechita et al. tested ten essential oils (basil, thyme, coriander, eucalyptus, lavender, lemon, fir tree, oregano, mint, and juniper) against *D. gallinae*, with the best results observed for lavender (>97% mortality after 48 and 72 h) and thyme (84% at 72 h) at a dose of 0.12 mg/cm^2^ (40). Furthermore, Ghrabi-Gammar et al. reported that essential oil from *Pelargonium graveolens* killed 100% of *D. gallinae* exposed to it for 24 h at a concentration of 0.21 mg/cm^2^ (41). Studies on plant extracts have shown that the acaricidal effect of plant oils with more chemical components is more significant than that of essential oils with fewer chemical components, possibly because of the synergistic effect between chemical components (42). Our study showed that star anise LC_90_+ motherwort LC_90_ had a significant acaricidal effect on nymphal and larval *D. gallinae*, with a mortality rate of 100.00%. Star anise contains total phenolics (64.00 mg/mL), total flavonoids (0.76 mg/mL) and tannins (0.12 mg/mL), all of which have well-known acaricide and insecticide activities (43, 44). For example, tannin has a strong protein precipitation capacity and a high oxidation activity, which can interfere with egg hatching and larval development and larval movement, as indicated by its acaricidal activity against larvae and eggs reported that phenolic compounds were very effective in killing adult *Rhipicephalus microplus* and inhibiting their oviposition and hatching, with methyl eugenol being more active in terms of disrupting egg hatching (45, 46). At present, the author has not found any information about motherwort with mite prevention. However, total phenols (16.70 mg/mL) and total flavonoids (0.20 mg/mL) were detected in the alcohol extract of motherwort in this study. Thus, the acaricidal properties of star anise LC_90_+ motherwort LC_90_ might result from the combined involvement of phenolics, tannins, and flavonoids. However, further studies are required to determine the mechanism of action behind these effects.

## Conclusion

In conclusion, star anise LC_90_+ motherwort LC_90_, rich in tannins, flavonoids, and polyphenol compounds, can be considered as a potential alternative to chemical insecticides because of their appreciable acaricidal properties against *D. gallinae*, an ectoparasite of veterinary importance in poultry and other livestock. As a continuation of this work and to confirm the current findings, the mechanism(s) of the synergistic and antagonistic effects between the herbs used in this study should be analyzed. In addition, examination of their acaricidal activities against ectoparasites should also be performed in field trials.

## Data availability statement

The original contributions presented in the study are included in the article/supplementary material, further inquiries can be directed to the corresponding author.

## Ethics statement

This study involved *in vitro* experiments and the Ethics governing the use and conduct of experiments on animals were strictly observed. Proper permits and consent were obtained from the Tongxu Poultry Farm management team before the *Dermanyssus gallinae* samples from poultry were used for this experiment.

## Author contributions

YJ: methodology, formal analysis, validation, and writing—original draft preparation. FJ: conceptualization, supervision, project administration, and writing—review and editing. HY, DL, and QG: investigation. XL, SZ, CN, and LZ: theoretical guidance, supervision, and analysis of the experimental results. All authors contributed to the article and approved the submitted version.

## Funding

This work was supported by the China Agriculture Research System of MOF and MARA (CARS-38).

## Conflict of interest

Author QG was employed by Hennan Hemu Animal Pharmaceutical Co., Ltd. The remaining authors declare that the research was conducted in the absence of any commercial or financial relationships that could be construed as a potential conflict of interest.

## Publisher's note

All claims expressed in this article are solely those of the authors and do not necessarily represent those of their affiliated organizations, or those of the publisher, the editors and the reviewers. Any product that may be evaluated in this article, or claim that may be made by its manufacturer, is not guaranteed or endorsed by the publisher.
